# London to Be Ambulanced

**Published:** 1889-03-30

**Authors:** 


### The Hospital

A Weekly Institutional Journal of

**Science, Medicine, Nursing, and Philanthropy.**

*For Hospitals, Asylums, Medical Practitioners, Students, Nurses, Families, Parishes Congregations, and the Charitable Public.*

Vol. V. No. 131.] [March 30, 1889.

### London to be Ambulanced.

London is the largest city in the world, and the richest, and the proudest; it ought by virtue of its elevation to be also the completest in every department of its public work. *Noblesse oblige* applies to towns and cities as well as to individuals and families. All genuine Londoners, whether "born" or "made," are decided admirers of their city, and keenly appreciate its honour or its disgrace. Our seventy hospitals, with their incomparable work, constitute a crown of bays, of which any queen of cities might be justly proud. In founding these, and raising them to their present standard of splendid excellence, the citizens of London have given evidence of qualities that adorn the character of man. In facilities for the kind and skilful treatment of the sick, our vast metropolis is almost as well provided as it is possible for a state or a town to be.

It must be clear as noon-day to all men that in a large city like ours, with its vast population, its enormous number of sick poor whose homes are such that removal from them to the hospital is the first condition of restoration to health, its crowded and rushing streets which produce accidents in daily crops all the year round, in such a "weltering mass" of confused humanity, some prompt, safe, and easy method of removing the sick and the injured to the hospitals is practically as necessary as the hospitals themselves. It is equally clear that no vehicles or litters used for the conveyance of fever and other infectious cases can with safety be employed for ordinary sick persons and those injured by accident or otherwise. As a matter of fact the safe and easy removal of persons suffering from infectious diseases is already provided for by the Metropolitan Asylums Board. The two classes, therefore, that have to be dealt with are those suffering from non-infectious diseases, and those injured in the street or other accidents.

At the meeting of The Hospitals Association held a few days ago, at which it was finally resolved to establish an ambulance scheme for the whole of London, Mr. Thomas Ryan, Secretary to St. Mary's Hospital, read a paper which dealt very exhaustively with the subject. For full details the paper itself must be consulted, and it may readily be obtained by application to the Secretary of The Hospitals Association, Norfolk Street, Strand. It seems that though the Metropolitan Asylums Board, which deals exclusively with paupers, has made ample provision for the removal of infectious cases to its infirmaries, it has by no means excelled in kindness towards those that are not infectious. Mr. Ryan states that the provision made by the parish authorities is in many cases defective in character or deficient in extent, and that in some instances no provision is made of any kind.

But by far the most numerous and important class of cases, from a street ambulance point of view, are of course street accidents and sudden illness. Mr. Ryan, from his experience at one of the large London hospitals, is well able to give a graphic picture of the circumstances of an ordinary street accident of the graver kind. He takes the not uncommon case of a man who has fallen from a scaffold erected outside a house and lighted upon the railings in front. The poor fellow has sustained, besides other injuries, a large jagged wound in the thigh, and the femoral artery has been torn through. A police-constable is speedily on the spot, and at once proceeds to render "first aid" according to the instructions systematically given to the Metropolitan Police by the St. John's Association. But in spite of this promptitude the injured man has necessarily lost a great deal of blood. Now comes the question of removal to the nearest hospital. It may be a mile off, or it may be even two. The man is in imminent danger of bleeding to death. He must, whatever happens, be got to the hospital at once. The constable often dare not wait for an ambulance litter, since according to present arrangements it may be twelve or fifteen minutes, or even half an hour before it can arrive. He is obliged to do the only practicable thing, and to call a cab. He drives off at once to the hospital, the patient sitting more or less upright on the seat. As the result of all this, the injured man is found on arrival in a state of syncope, and dies within a few minutes of his admission. This is affirmed to be a typical case, and was selected by Mr. Ryan because such an instance actually occurred at his own hospital a very short time ago.

It will scarcely be believed, but the only systematic provision now existing for the removal of persons injured in the London streets to their homes or to hospitals is that administered by the Metropolitan Police Force. At every police-station within a radius of four miles from Charing Cross, a wheeled litter of the St. John's Ambulance pattern is kept. There are forty-nine such "Police Ambulance Stations," scattered at very unequal distances over the area of fifty square miles contained within the four-mile circle. In order to supply the metropolis with ambulance accommodation which shall be really adequate, seventy additional stations with a wheeled litter at each are immediately required.

That seems undoubtedly a large and costly demand; but Mr. Ryan proposes to utilize as ambulance centres, not only the police stations, but the stations of the Fire Brigade, various railway stations, and also the hospitals themselves. Taking these four classes of buildings it is found that not only are there sufficient stations in existence, but that they are suitably distributed all over London; and what is most to the point, that they are immediately available. The Fire Brigade and the officials of the different railway companies have given their fullest sympathy to Mr. Ryan's very practical proposals.

The only question that remains for consideration is that of cost; and here the public will be met by a most welcome surprise. When the founding and furnishing of seventy new ambulance stations is mentioned, the mind at once begins to think of the thousands and tens of thousands of pounds which such an enormous scheme will involve. But nothing of the kind. The stations are already in existence, and the seventy two-wheel Ashford litters, with which it is proposed to equip them, can be supplied at a cost of £11 each. There will be other small preliminary expenses, but it is estimated that with rigid economy the total outlay for the complete equipment of the whole of the new ambulance centres need not exceed £1,500. That sum has already been promised to Mr Burdett by a few sympathisers. Of course, for the proper conduct of all the ambulances, for their superintendence, repair, and other necessary work, a certain annual expenditure will also be involved; but even that, it is believed, need not exceed more than about £300 per annum; and for that sum we appeal with all confidence to the public on behalf of this new and most important departure. We venture to think that for urgency of need, for simplicity of plan, for inexpensiveness and for practical utility, no philanthropic scheme which was ever proposed better deserved the prompt sympathy and help of a discriminating and charitable press and public. Any subscriptions forwarded to us will be duly acknowledged.

